# The combined effect of visit-to-visit variability in HbA1c and systolic blood pressure on the incidence of cardiovascular events in patients with type 2 diabetes

**DOI:** 10.1136/bmjdrc-2015-000129

**Published:** 2015-11-13

**Authors:** Toshiko Takao, Yutaka Matsuyama, Machi Suka, Hiroyuki Yanagisawa, Yasuhiko Iwamoto

**Affiliations:** 1 Division of Diabetes and Metabolism, The Institute for Adult Diseases, Asahi Life Foundation, Tokyo, Japan; 2 Department of Biostatistics, School of Public Health, University of Tokyo, Tokyo, Japan; 3 Department of Public Health and Environmental Medicine, The Jikei University School of Medicine, Tokyo, Japan

**Keywords:** Cardiovacsular Disease(s), HbA1c, Blood Pressure, Type 2 Diabetes

## Abstract

**Objective:**

To investigate the association between long-term visit-to-visit variability in glycated hemoglobin (HbA1c) and systolic blood pressure (SBP) and the incidence of cardiovascular disease (CVD) in patients with type 2 diabetes.

**Methods:**

We retrospectively enrolled 632 patients with type 2 diabetes and no history of CVD who first visited our hospital between 1995 and 1996, were followed-up for ≥1 year, attended at least 4 clinic visits and had at least 1 visit per year. Patients were followed until June 2012 at the latest, and mailed questionnaires.

**Results:**

During the median follow-up period (15.4 years), 81 patients developed CVD. Multivariate analysis revealed that the coefficient of variation (CV) and the variation independent of mean (VIM) for HbA1c and SBP were significant predictors of CVD incidence independent of mean HbA1c and SBP. Patients were classified into 4 groups by median HbA1cCV and SBPCV values and by median HbA1cVIM and SBPVIM values. Among these groups, the HRs were highest in the high-HbA1cCV/high-SBPCV and high-HbA1cVIM/high-SBPVIM groups and were significantly higher compared with those in the low-HbA1cCV/low-SBPCV and low-HbA1cVIM/low-SBPVIM groups, respectively. Among patients with mean SBP≥130 mm Hg, the HRs associated with HbA1cCV and HbA1cVIM were drastically elevated compared with those with mean SBP<130 mm Hg (interaction p<0.05).

**Conclusions:**

Long-term visit-to-visit variability in HbA1c and SBP represented a combined and additive risk for CVD incidence in patients with type 2 diabetes. It is suggested that a synergistic effect exists between HbA1c variability and mean SBP levels for CVD incidence.

**Key messages**

- Using a database comprising ‘real-world’ observations with a long-term follow-up period, the present study revealed that long-term visit-to-visit variability in glycated hemoglobin (HbA1c) and systolic blood pressure (SBP) represented a combined and additive risk for cardiovascular disease (CVD) incidence in patients with type 2 diabetes.
- It is suggested that a synergistic effect exists between HbA1c variability and mean SBP levels for the incidence of CVD. In addition, SBP variability can be a risk factor for CVD incidence, even if the mean SBP is maintained within the normal range.
- Our findings indicate the possibility that stabilization of variability in HbA1c and SBP as well as lowering of their mean levels can be an efficient strategy for preventing the incidence of CVD.

## Introduction

Recent clinical evidence has raised the possibility that visit-to-visit glycated hemoglobin (HbA1c) variability^1–4^ and blood pressure (BP) variability^5–7^ independently predict macrovascular complications and/or all-cause mortality in patients with type 2 diabetes. However, to date, no study has examined the combined risk associated with visit-to-visit variability in HbA1c and systolic BP (SBP) simultaneously. In addition, the differences in the effects between HbA1c variability and SBP variability on the incidence of cardiovascular disease (CVD) according to the mean HbA1c and SBP values have been scarcely investigated.

Basic research data have shown that glucose fluctuations can cause oxidative stress,^8–10^ chronic inflammation, and endothelial dysfunction, which are involved in the progression of atherosclerosis.^11^ ^12^ Additionally, increased BP variability may reflect arterial stiffness and baroreceptor dysfunction, which have been associated with arteriosclerosis and can result in cardiovascular events.^13–17^ However, the precise mechanisms have not been fully elucidated.

In this study, we evaluated the combined effect of visit-to-visit variability in HbA1c and SBP on the incidence of CVD using a database of ‘real-world’ observations with long-term follow-up in patients with type 2 diabetes. In addition, we analyzed the differences in the effects between HbA1c variability and SBP variability on the incidence of CVD according to the mean HbA1c and SBP values.

## Methods

### Study participants

Of the 1912 patients who first visited the outpatient clinic of our hospital from January 1995 to December 1996, we retrospectively recruited 632 patients with type 2 diabetes who attended at least four clinic visits, with at least one clinic visit per year, and had been followed up for ≥1 year. Patients were excluded if they had impaired glucose tolerance or a history of CVD at the first visit or within 1 year thereafter. They were followed for the incidence of CVD until June 2012.

Of the 632 patients, 293 (46.4%) completed the follow-up, and 26 (4.1%) died. In June 2012, a questionnaire was mailed to the remaining 313 patients (49.5%) who had transferred to other hospitals or dropped out. One hundred and thirty-six (21.5%) responses were obtained. Of these, 27 deaths were confirmed. However, there were no responses from 177 patients (28.0%) who were regarded as censored cases at the last visit. Finally, the overall follow-up rate was 72.0% (455/632).

The following baseline characteristics of the patients were analyzed: age, sex, diabetes duration, BP, body mass index (BMI), HbA1c level, serum lipid level, serum creatinine (SCr) level, estimated glomerular filtration rate (eGFR), smoking status, alcohol intake, diabetes therapy, use of antihypertensive agents (ACE inhibitor, calcium channel blocker, α-blocker, or β-blocker), and/or use of a lipid-lowering drug. The merely renin-angiotensin system inhibitors available in Japan in 1995–1996 were ACE inhibitors. Initial therapy was defined as treatment started before the first visit, at the first visit, or within 6 months thereafter. Patients who received a combination of insulin and an oral antidiabetic drug were considered as insulin-treated patients.

The study design was consistent with the Japanese government's Ethical Guidelines Regarding Epidemiological Studies and was in accordance with the Declaration of Helsinki. The protocol of this study was reviewed and approved by our Institutional Review Board and informed consent was obtained from all enrolled patients.

### End point definition

The end point was the first CVD event, defined as fatal or non-fatal acute myocardial infarction, coronary artery procedure (bypass surgery or angioplasty), or stroke (ischemic or hemorrhagic), that required hospitalization. These events were determined according to a thorough review of medical records and responses of the questionnaires. Patients who had no CVD event, including those who had died from all other causes except CVD, were considered censored cases at the last clinic visit.

### Data collection and variables determined

Capillary blood was drawn at each visit to determine blood glucose and HbA1c levels, irrespective of fasting or postprandial status. HbA1c levels were determined using an automated glycohemoglobin analyzer (Tosoh Bioscience, Tokyo, Japan); beginning in November 1994, HbA1c levels were determined using high-performance liquid chromatography, as standardized by the Japan Diabetes Society (JDS). HbA1c values obtained before January 2007 were converted to JDS standard values (reference range 4.3–5.8%) using linear regression equations. The equations were derived from duplicate assays using old and/or new devices or standard substances. Beginning in June 2012, we used the National Glycohemoglobin Standardization Program (NGSP)-certified method, and all earlier HbA1c (%) values were converted to NGSP values (%) using the following equation: (HbA1c (NGSP) (%)=1.02×HbA1c (JDS) (%)+0.25 (%)).^18^ The intrapersonal mean, coefficient of variation (CV), and variation independent of mean (VIM) of all recorded HbA1c measurements were calculated for each patient, and CV and VIM were employed as a measure of visit-to-visit variability in HbA1c.

BP was typically determined once at each visit in the sitting position by a trained medical technologist using an electronic sphygmomanometer (OMRON, Kyoto, Japan). The intrapersonal mean, CV, and VIM of all recorded SBP measurements were calculated for each patient, and CV and VIM were employed as a measure of visit-to-visit variability in SBP. The recorded BP values were used, in spite of whether the patient initiated or added an antihypertensive agent during the follow-up.

Lipids were measured irrespective of fasting or postprandial status. The total cholesterol (TC) level was determined using an enzymatic method. The high-density lipoprotein cholesterol (HDL-C) level was determined using a dextran sulfate and magnesium precipitation method until April 25, 1996, after which HDL-C was determined using a direct enzymatic method. HDL-C data from the precipitation method were converted to the direct enzymatic method equivalents using a linear regression equation derived from duplicate assays. The baseline TC: HDL-C ratio (TC/HDL-C) was employed as a covariate in the analysis because TC/HDL-C has been shown to be the best predictor of CVD among males with type 2 diabetes.^19^ ^20^ Moreover, TC/HDL-C was found to be a stronger predictor of CVD compared with non-HDL-C in the UK Prospective Diabetes Study (UKPDS) risk engine.^21^

The SCr level was determined using the Jaffe-Rate method until June 11, 1995, after which SCr was determined using an enzymatic method. SCr data obtained using the Jaffe-Rate method were converted to enzymatic method equivalents using a linear regression equation derived from duplicate assays. eGFR was determined using the following equation, as advocated by the Japanese Society of Nephrology: eGFR (mL/min/1.73 m^2^) =194×SCr^−1.094^×age^−0.287^ (×0.739 if female).^22^

### Statistical analysis

Data are expressed as means±SD for continuous variables or as numbers and percentages for categorical variables. Since the data distributions of the follow-up period and the number of visits were skewed, they were described as median values (IQR). Differences between patients who did and did not develop a CVD event were analyzed using Student's t test for continuous variables and the χ^2^ test or Fisher's exact test, as needed, for categorical variables. Age, sex, and diabetes duration were adjusted using logistic regression analysis.

The Kaplan-Meier survival curves for a CVD event were given for the four groups classified by median HbA1cCV and SBPCV values, after adjusting for age, mean HbA1c, mean SBP, and the number of visits. Values for the number of visits were ln-transformed for inclusion in the model to adjust for the possibility that the number of visits could influence variability.

Multivariate analyses were performed using Cox proportional hazard models to evaluate the respective and combined effects of visit-to-visit variability in HbA1c and SBP as continuous variables on the incidence of CVD. The analysis was performed after adjusting for mean HbA1c, mean SBP, the number of visits (ln-transformed), age, sex, diabetes duration, BMI, TC/HDL-C, eGFR, baseline smoking status, baseline alcohol intake, baseline use of insulin, and baseline use of an antihypertensive agent. HRs are reported in 1SD increments.

The combined effects of the visit-to-visit variability in HbA1c and SBP on the incidence of CVD as categorical variables were analyzed. Patients were classified into four groups by median HbA1cCV and SBPCV values. HRs for the incidence of CVD associated with these four groups (with lower HbA1cCV and lower SBPCV serving as the reference group) were calculated using a Cox proportional hazard model after adjusting for the aforementioned covariates. Similarly, the analysis was also performed in respect of the four groups classified by median HbA1cVIM and SBPVIM values (with lower HbA1cVIM and lower SBPVIM serving as the reference group).

Furthermore, a stratified analysis was performed according to the mean HbA1c and SBP levels of 7.0% and 130 mm Hg, respectively, and the effects of visit-to-visit variability in HbA1c and SBP on the incidence of CVD were evaluated as continuous variables using multivariate Cox proportional hazard models after adjusting for the aforementioned covariates. The interaction was examined.

The SAS V.9.4 software package (SAS Institute, Cary, North Carolina, USA) was used for all statistical analyses. Two-tailed p values <0.05 were considered to indicate significance.

## Results

The baseline clinical characteristics of all patients classified according to the incidence of CVD events during follow-up are shown in table 1. During the follow-up period, 81 (12.8%) patients (65 males and 16 females) had suffered a CVD event. After adjusting for age, sex, and diabetes duration, patients who had suffered a CVD event were significantly older, had a significantly longer diabetes duration, a significantly higher TC level, and a significantly lower HDL-C level. In addition, patients with CVD were significantly more likely to be taking an antihypertensive agent (ACE inhibitor, calcium channel blocker, or β-blocker) and a lipid-lowering agent compared with those without CVD.

**Table 1:** Baseline characteristics of all patients following classification according to the incidence of CVD during follow-up

	All	CVD event		p Value	Adjusted p value*
		No event	Event		
n	632	551	81		
Male (%)	519 (82.1)	454 (82.4)	65 (80.3)	0.638	0.997
Age (years)	55.7±9.3	55.2±9.3	58.8±8.7	0.001	0.030
Duration of diabetes (years)	5.7±6.7	5.3±6.4	8.5±8.2	0.001	0.003
BMI (kg/m^2^)	23.3±3.3	23.3±3.3	23.6±3.1	0.506	0.169
HbA1c (%)	8.0±1.7	8.0±1.7	8.2±1.6	0.235	0.287
(mmol/mol)	64.2±18.7	63.9±18.9	66.5±17.9	0.235	0.287
SBP (mm Hg)	133.4±21.1	132.6±20.6	138.8±23.3	0.014	0.071
DBP (mm Hg)	77.7±12.6	77.5±12.4	79.6±13.8	0.160	0.128
TC (mg/dL)	209.5±37.6	208.0±36.6	219.7±42.6	0.008	0.011
HDL-C (mg/dL)	49.9±12.8	50.3±13.0	46.9±11.5	0.026	0.002
eGFR (mL/min/1.73 m^2^)	79.8±18.6	80.1±18.5	77.8±19.5	0.306	0.858
Current smoker	267 (42.3)	235 (42.7)	32 (39.5)	0.593	0.889
Alcohol intake	477 (75.5)	422 (76.6)	55 (67.9)	0.090	0.232
Initial therapies					
Oral antidiabetic drugs†	263 (41.6)	222 (40.3)	41 (50.6)	0.078	0.240
Insulin‡	83 (13.1)	72 (13.1)	11 (13.6)	0.898	0.598
Antihypertensive agents	138 (21.8)	109 (19.8)	29 (35.8)	0.001	0.023
ACE inhibitors	54 (8.6)	39 (7.1)	15 (18.5)	0.0006	0.004
Calcium channel blockers	102 (16.2)	77 (14.0)	25 (30.9)	0.0001	0.006
β-blockers	19 (3.0)	12 (2.2)	7 (8.6)	0.006	0.003
α-blockers	7 (1.1)	6 (1.1)	1 (1.2)	1.000	0.837
Lipid-lowering agents	68 (10.8)	53 (9.6)	15 (18.5)	0.016	0.045

Values are numbers (percentages) or means±SDs.

*Age, sex, and diabetes duration-adjusted p value. Age, sex, and diabetes duration were adjusted except for itself, respectively.

†Excludes patients treated with oral antidiabetic drugs and insulin.

‡Includes patients treated with oral antidiabetic drugs and insulin.

BMI, body mass index; CVD, cardiovascular disease; DBP, diastolic blood pressure; eGFR, estimated glomerular filtration rate; HbA1c, glycated hemoglobin; HDL-C, high-density lipoprotein cholesterol; SBP, systolic blood pressure; TC, total cholesterol.

The total number of visits was 55 855 (per-patient median, 81; IQR 36–126.5). The median follow-up period was 15.4 years (IQR 6.6–16.4).

### Correlations between variability in HbA1c and SBP, as well as variability and mean HbA1c or SBP

HbA1cCV was correlated with mean HbA1c (r=0.428, p<0.0001). However, no correlation was observed between SBPCV and mean SBP (r=0.038, p=0.341). HbA1cVIM (proportional to SD/mean^2.70^) was independent of mean HbA1c (r=−0.0006, p=0.989), and SBPVIM (proportional to SD/mean^1.11^) was independent of mean SBP (r=−0.00008, p=0.998). A weak correlation was observed between HbA1cCV and SBPCV (r=0.107, p=0.007), as well as between HbA1cVIM and SBPVIM (r=0.100, p=0.012), which were statistically significant owing to the large number of patients involved.

### Adjusted Kaplan-Meier survival curves for a CVD event

**Figure 1:**
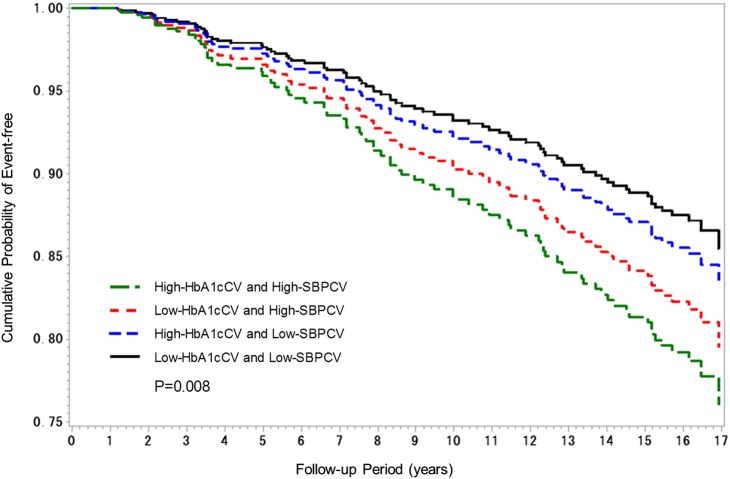
Kaplan-Meier survival curves for a CVD event classified according to the median HbA1cCV and SBPCV values, after adjusting for age, mean HbA1c, mean SBP, and the number of visits (ln-transformed) (CV, coefficient of variation; CVD, cardiovascular disease; HbA1c, glycated hemoglobin; SBP, systolic blood pressure).

In figure 1, the Kaplan-Meier survival curves for a CVD event were given for the four groups classified by median HbA1cCV and SBPCV values, after adjusting for age, mean HbA1c, mean SBP, and the number of visits (ln-transformed). The adjusted survival curves revealed a clear association between variability in HbA1c and SBP and the incidence of CVD. The highest incidence was observed in the high-HbA1cCV and high-SBPCV group, followed by the low-HbA1cCV and high-SBPCV group, and finally the high-HbA1cCV and low-SBPCV group. Conversely, the lowest incidence was observed in the low-HbA1cCV and low-SBPCV group.

### Respective and combined effects of visit-to-visit variability in HbA1c and SBP on the incidence of CVD

The respective and combined effects of HbA1cCV and SBPCV as continuous and categorical variables on the incidence of CVD were evaluated by a multivariate analysis performed using a Cox proportional hazard model (table 2). The analysis was performed after adjusting for the patient characteristics described in the Methods section. The HRs were calculated in accordance with a 1SD increment. In model 1, HbA1cCV but not SBPCV was incorporated as one of the covariates in combination with other clinical variables. In model 2, SBPCV but not HbA1cCV was incorporated. In model 3, both of these were incorporated. In these three models, HbA1cCV and SBPCV were treated as a standardized continuous variable. In model 4, HbA1cCV and SBPCV were also included in the model as model 3, but these were treated as categorical variables, being dichotomized by the respective median value.

**Table 2:** Multivariate Cox proportional hazard models for the incidence of CVD in association with HbA1cCV and SBPCV as continuous and categorical variables divided by their respective median values

	Model 1		Model 2		Model 3		Model 4	
	HR (95% CI)	p Value	HR (95% CI)	p Value	HR (95% CI)	p Value	HR (95% CI)	p Value
Continuous variables								
HbA1cCV (1SD increment)	1.39 (1.10 to 1.76)	0.006	NA	NA	1.33 (1.04 to 1.70)	0.024	NA	NA
SBPCV (1SD increment)	NA	NA	1.33 (1.07 to 1.63)	0.009	1.26 (1.02 to 1.57)	0.032	NA	NA
Categorical variables								
Low HbA1cCV and low SBPCV	NA	NA	NA	NA	NA	NA	1	
Low HbA1cCV and high SBPCV	NA	NA	NA	NA	NA	NA	2.55 (1.22 to 5.32)	0.013
High HbA1cCV and low SBPCV	NA	NA	NA	NA	NA	NA	2.64 (1.16 to 6.00)	0.020
High HbA1cCV and high SBPCV	NA	NA	NA	NA	NA	NA	3.08 (1.45 to 6.55)	0.003

All models were adjusted for mean HbA1c, mean SBP, number of visits (ln-transformed), age, sex, diabetes duration, BMI, TC/HDL-C, eGFR, baseline smoking status, baseline alcohol intake, baseline use of insulin, and baseline use of antihypertensive agents.

BMI, body mass index; CV, coefficient of variation; CVD, cardiovascular disease; eGFR, estimated glomerular filtration rate; HbA1c, glycated hemoglobin; HDL-C, high-density lipoprotein cholesterol; NA, not applicable; SBP, systolic blood pressure; TC, total cholesterol.

In model 1, HbA1cCV was a significant predictor of the incidence of CVD independent of mean HbA1c. In model 2, SBPCV was a significant predictor of the incidence of CVD independent of mean SBP. In model 3, HbA1cCV and SBPCV as continuous variables were significant predictors of the incidence of CVD, independent of mean HbA1c and mean SBP, simultaneously. In model 4, patients were classified into four groups by median HbA1cCV and SBPCV values. HRs for the incidence of CVD associated with these four groups (with low-HbA1cCV and low-SBPCV serving as the reference group) were calculated. The HRs were highest in the high-HbA1cCV and high-SBPCV group and significantly higher in the low-HbA1cCV and high-SBPCV group and high-HbA1cCV and low-SBPCV group than in the low-HbA1cCV and low-SBPCV group.

In table 3, VIM was used instead of CV in all models shown in table 2. The results of model 1, 2, and 3 of table 3 were similar to those of table 2. In model 4, the HRs were significantly higher in the high-HbA1cVIM and high-SBPVIM group, and higher, but not significantly, in the low-HbA1cVIM and high-SBPVIM group and high-HbA1cVIM and low-SBPVIM group than in the low-HbA1cVIM and low-SBPVIM group.

**Table 3:** Multivariate Cox proportional hazard models for the incidence of CVD in association with HbA1cVIM and SBPVIM as continuous and categorical variables divided by their respective median values

	Model 1		Model 2		Model 3		Model 4	
	HR (95% CI)	p Value	HR (95% CI)	p Value	HR (95% CI)	p Value	HR (95% CI)	p Value
Continuous variables								
HbA1cVIM (1SD increment)	1.33 (1.07 to 1.65)	0.011	NA	NA	1.28 (1.02 to 1.61)	0.035	NA	NA
SBPVIM (1SD increment)	NA	NA	1.33 (1.08 to 1.63)	0.008	1.27 (1.03 to 1.57)	0.026	NA	NA
Categorical variables								
Low HbA1cVIM and low SBPVIM	NA	NA	NA	NA	NA	NA	1	
Low HbA1cVIM and high SBPVIM	NA	NA	NA	NA	NA	NA	1.77 (0.90 to 3.51)	0.10
High HbA1cVIM and low SBPVIM	NA	NA	NA	NA	NA	NA	1.72 (0.82 to 3.63)	0.15
High HbA1cVIM and high SBPVIM	NA	NA	NA	NA	NA	NA	2.19 (1.12 to 4.29)	0.022

All models were adjusted for mean HbA1c, mean SBP, number of visits (ln-transformed), age, sex, diabetes duration, BMI, TC/HDL-C, eGFR, baseline smoking status, baseline alcohol intake, baseline use of insulin, and baseline use of antihypertensive agents.

BMI, body mass index; CVD, cardiovascular disease; eGFR, estimated glomerular filtration rate; HbA1c, glycated hemoglobin; HDL-C, high-density lipoprotein cholesterol; NA, not applicable; SBP, systolic blood pressure; TC, total cholesterol; VIM, variation independent of mean.

### Stratified analysis by mean HbA1c and SBP levels and the effects of visit-to-visit variability in HbA1c and SBP, as continuous variables, on the incidence of CVD

A stratified analysis was performed by mean HbA1c and SBP levels of 7.0% and 130 mm Hg, respectively (table 4). The effects of visit-to-visit variability in HbA1c and SBP on the incidence of CVD were evaluated as continuous variables using multivariate Cox proportional hazard models. The covariates are described in the Methods section. In models 1 and 2, for the stratum of patients with mean HbA1c<7.0%, neither HbA1cCV nor HbA1cVIM was significant, but SBPCV and SBPVIM were borderline significant. For the stratum of patients with mean HbA1c≥7.0%, HbA1cCV and HbA1cVIM were borderline significant, whereas neither SBPCV nor SBPVIM was significant. For the stratum of patients with mean SBP<130 mm Hg, neither HbA1cCV nor HbA1cVIM was significant, whereas SBPCV and SBPVIM were significant. For the stratum of patients with mean SBP≥130 mm Hg, the HRs associated with HbA1cCV and HbA1cVIM were drastically elevated compared with those for the stratum of patients with mean SBP<130 mm Hg (interaction p=0.018 for HbA1cCV; interaction p=0.016 for HbA1cVIM). Thus, HbA1cCV and HbA1cVIM were significant predictors, whereas neither SBPCV nor SBPVIM was significant.

**Table 4:** HRs for incidence of CVD associated with variability in HbA1c and SBP stratified according to mean HbA1c and SBP levels

	Mean HbA1c<7.0%		Mean HbA1c≥7.0%		Interaction p	Mean SBP<130 mm Hg		Mean SBP≥130 mm Hg		Interaction p
	HR (95% CI)	p Value	HR (95% CI)	p Value		HR (95% CI)	p Value	HR (95% CI)	p Value	
Events/patients	37/338		44/294			37/306		44/326		
Model 1										
HbA1cCV (1SD increment)	1.20 (0.76 to 1.88)	0.44	1.37 (1.00 to 1.87)	0.052		0.99 (0.65 to 1.52)	0.97	1.77 (1.28 to 2.46)	0.0007	0.018
SBPCV (1SD increment)	1.33 (0.97 to 1.81)	0.076	1.30 (0.89 to 1.88)	0.18	0.79	1.59 (1.11 to 2.29)	0.013	1.16 (0.86 to 1.56)	0.34	
Model 2										
HbA1cVIM (1SD increment)	1.17 (0.83 to 1.66)	0.37	1.38 (0.99 to 1.93)	0.054		0.96 (0.64 to 1.44)	0.85	1.73 (1.25 to 2.38)	0.0009	0.016
SBPVIM (1SD increment)	1.32 (0.97 to 1.80)	0.076	1.31 (0.90 to 1.90)	0.16	0.82	1.59 (1.12 to 2.28)	0.011	1.19 (0.89 to 1.60)	0.25	

All models were adjusted for mean HbA1c, mean SBP, number of visits (ln-transformed), age, sex, diabetes duration, BMI, TC/HDL-C, eGFR, baseline smoking status, baseline alcohol intake, baseline use of insulin, and baseline use of antihypertensive agents.

BMI, body mass index; CV, coefficient of variation; CVD, cardiovascular disease; eGFR, estimated glomerular filtration rate; HbA1c, glycated hemoglobin; HDL-C, high-density lipoprotein cholesterol; SBP, systolic blood pressure; TC, total cholesterol; VIM, variation independent of mean.

## Discussion

Long-term visit-to-visit variability in HbA1c and SBP represented a combined and additive risk for the incidence of CVD simultaneously in patients with type 2 diabetes. In addition, the risk of CVD associated with an increase in HbA1c variability was drastically elevated among patients with mean SBP≥130 mm Hg. In contrast, the CVD risk associated with an increase in SBP variability increased significantly among patients with mean SBP<130 mm Hg, whereas an increase in HbA1c variability was likely to have no effect. It is suggested that a synergistic effect exists between HbA1c variability and mean SBP levels for the incidence of CVD (interaction p<0.05).

The results of the Action in Diabetes and Vascular Disease: Preterax and Diamicron Modified Release Controlled Evaluation (ADVANCE) trial showed that visit-to-visit SBP variability is an independent risk factor for macrovascular complications in patients with type 2 diabetes.^6^ Recently, the same trial, as the first large-scale study, showed that visit-to-visit HbA1c variability predicts the future development of macrovascular events independent of cardiovascular risk factors, including mean HbA1c.^4^ However, the study differed from this study in that participants had type 2 diabetes, were ≥55 years old, and had a history of major macrovascular or microvascular disease or at least one other risk factor for vascular disease. Furthermore, no data were reported on the combined effect of visit-to-visit variability in HbA1c and SBP in that trial. Thus, this study is the first to report the combined effect of visit-to-visit variability in HbA1c and SBP on the incidence of CVD simultaneously in patients with type 2 diabetes.

The relationship between BP variability, calculated for different long-term sequential time frames, and mortality risk was reported.^23^ It was a large cohort study using real-world clinical BP data of 14 522 treated patients with hypertension who were followed up over 35 years. The results indicated that long-term variability in SBP and diastolic BP (DBP) calculated for the long term (1–4 years) and ultra long term (5–9 years) were significant predictors of mortality, independent of mean BP. This relationship was also evident in subgroups with mean SBP<140 mm Hg, which agrees with our results. In our study, a stratified analysis by mean SBP of 130 mm Hg was also performed. HbA1c variability was a significant predictor among patients with mean SBP≥130 mm Hg, but SBP variability was not. In contrast, SBP variability was a significant predictor, but HbA1c variability was not among patients with mean SBP<130 mm Hg. Thus, even if mean SBP was maintained within the normal range, increased SBP variability was a risk factor for a CVD event.

We previously reported the relationships between the risk of CVD in patients with type 2 diabetes and both visit-to-visit variability and time-to-effect differences in BP.^7^ Our earlier study showed that increases in SBP over the preceding 3–5 years resulted in a significant CVD risk. Therefore, increased HbA1c variability over the preceding 3–5 years could emerge as a more harmful risk factor for the incidence of CVD. Thus, stabilizing variability in HbA1c level and lowering BP during these periods seem to be particularly important.

Glycemic variability is associated with a risk of severe hypoglycemia.^24^ Severe hypoglycemia is also associated with a higher risk of CVD.^25^ In our study, however, no information about a severe hypoglycemic episode in which a patient required the assistance of another person was available. Therefore, hypoglycemia was defined by a fasting or casual blood glucose level at clinic visits of less than 60 mg/dL at least once during follow-up. Hypoglycemia occurred in 32 patients. There was no association between hypoglycemia at clinic visits and the incidence of CVD (data not shown). Furthermore, the association between visit-to-visit variability in HbA1c and SBP and the incidence of CVD was independent of hypoglycemia at clinic visits (data not shown). Even if hypoglycemia was defined as less than 50 mg/dL, results remained almost unchanged (data not shown).

The possible practical factors, such as age, the number of visits, seasonal changes, lifestyle factors, non-adherence with antidiabetic and antihypertensive medications, and improper titration/dosing of those medications, could contribute to visit-to-visit variability in HbA1c and SBP. In this study, we evaluated what baseline characteristics contributed to subsequent visit-to-visit variability in HbA1c and SBP. Younger age and increased baseline HbA1c contributed to HbA1c variability, while older age, increased baseline SBP, decreased baseline BMI and eGFR, baseline use of insulin, and baseline use of antihypertensive agents contributed to SBP variability (data not shown).

SD as a measure of variability is most familiar to clinicians and easy to calculate, although SD is affected by the mean value. The same analyses were also performed using SD; consequently, similar results were obtained. Concretely, in the participants of our study, the median values (IQR) of the mean HbA1c (%), HbA1cSD (%), mean SBP (mm Hg), and SBPSD (mm Hg) were 6.93 (6.43–7.48), 0.57 (0.38–0.79), 130.6 (121.8–139.5), and 11.6 (9.8–14.1), respectively. The risk of the incidence of CVD significantly increased 2.45-fold for each 1% increase in HbA1cSD and 2.02-fold for every 10 mm Hg increase in SBPSD. It is important that clinicians pay attention to variability in HbA1c and SBP, which may represent a higher cardiovascular risk than their mean values and can lead to combined additive risk for the incidence of CVD.

One strength of this study was the use of a database comprising ‘real-world’ observations with a long-term follow-up period. In addition, we addressed a topic that may help provide novel and effective strategies for preventing CVD in patients with type 2 diabetes. However, several limitations must also be mentioned. First, this is a retrospective observational cohort study. The results merely indicate the association, but not causation. In addition, potential information biases included changes in sample examination methods with time and differences in the number of visits. However, some data generated by the different measurement methods were converted using linear regression equations derived from duplicate assays. Visit-to-visit BP variability increases with the number of visits.^26^ To adjust for the possibility that the number of visits could influence variability, the number of visits was included in the model as a covariate after being ln-transformed. Second, BP data were derived typically from a single measurement obtained at each visit. However, visit-to-visit BP variability is a reproducible, not random, phenomenon.^27^ ^28^ It can be assumed that higher reproducibility is achieved when automated devices are used.^27–29^ Third, the incidence of CVD was partly self-reported by patients. However, the end point was defined clearly, and 66 (81.5%) of the 81 CVD events were determined according to a thorough review of medical records. Therefore, merely 15 events (18.5%) were based on results of the questionnaire. Fourth, lipids were determined irrespective of fasting or postprandial status. Therefore, we could not conduct an analysis using triglyceride. Nevertheless, TC/HDL-C was used as a covariate for the analysis, because TC/HDL-C is the best lipid predictor of CVD for males with type 2 diabetes.^19^ ^20^ Fifth, non-adherence with antidiabetic and antihypertensive medications could contribute to visit-to-visit variability in HbA1c and SBP; however, we have no data on adherence with medication. Finally, our study participants were recruited from a single hospital in Japan and included more males than females; however, their clinical characteristics were similar to those of patients in another large-scale study in Japan.^30^ It is uncertain whether our findings can be generalized to other ethnic groups. Prospective international multicenter trials are needed.

In conclusion, long-term visit-to-visit variability in HbA1c and SBP represented a combined and additive risk for the incidence of CVD simultaneously in patients with type 2 diabetes. In addition, it is suggested that a synergistic effect exists between HbA1c variability and mean SBP levels for the incidence of CVD. Even if mean SBP is maintained within the normal range, SBP variability can be a risk factor for a CVD event. Our findings indicate the possibility that stabilization of variability in HbA1c and SBP as well as lowering of their mean levels can be an efficient strategy for preventing the incidence of CVD.
